# Intravascular large B-cell lymphoma as a covert trigger for hemophagocytic lymphohistiocytosis complicated with capillary leak syndrome: a case report and literature review

**DOI:** 10.3389/fimmu.2024.1403376

**Published:** 2024-07-12

**Authors:** Jingjing Wen, Juan Xu, Jie Ji, Wenyan Zhang, Qin Zheng, Ting Liu, Yuhuan Zheng, Hongbing Ma

**Affiliations:** ^1^ Department of Hematology/Institute of Hematology, West China Hospital, Sichuan University, Chengdu, China; ^2^ Department of Hematology, Mianyang Central Hospital, School of Medicine, University of Electronic Science and Technology of China, Mianyang, China; ^3^ Department of Pathology, West China Hospital, Sichuan University, Chengdu, China; ^4^ Department of Laboratory Medicine, West China Hospital, Sichuan University, Chengdu, China

**Keywords:** intravascular large B-cell lymphoma, capillary leak syndrome, hemophagocytic lymphohistiocytosis, cytokine, immunochemotherapy, transplantation

## Abstract

Intravascular large B-cell lymphoma (IVLBCL) is a rare subtype of non-Hodgkin lymphoma. Patients with hemophagocytic lymphohistiocytosis (HLH)-associated IVLBCL variants exhibit significantly poor survival. Cytokines play pivotal roles in malignancy-associated HLH as well as in capillary leak syndrome (CLS). The pathogenesis of CLS involves hyperpermeability and transient endothelial dysfunction. Here, we report the first case of HLH-associated IVLBCL variant complicated with CLS. The patient presented with fever, refractory hypoproteinemia, hypotension and severe edema, followed by telangiectasias. Treatment with etoposide and dexamethasone and hydroxyethyl starch-based artificial colloid led to transient improvement. The diagnosis of IVLBCL was confirmed after the sixth bone marrow biopsy. Subsequently, the R-CHOP (rituximab, cyclophosphamide, hydroxydaunorubicin, vincristine, and prednisolone) regimen was administered and resulted in prompt alleviation of CLS and HLH symptoms. The patient has survived for more than 6 years after combination of immunochemotherapy and autologous peripheral stem-cell transplantation. This case provides some insights into the mechanism and clinical management of IVLBCL complicated with HLH and CLS. Similar cases concerning lymphoma-associated CLSs were also reviewed.

## Introduction

Intravascular large B-cell lymphoma (IVLBCL) is a rare subtype of non-Hodgkin lymphoma, with an incidence rate of approximately 0.095/100,000 individuals (1). IVLBCL is characterized by the accumulation of neoplastic cells within the lumens of invariably small blood vessels, particularly capillaries and postcapillary venules (2). The invasion of blood vessels in various organs throughout the body by tumor cells leads to vascular obstruction, resulting in a complex and variable array of symptoms. The presentation of symptoms is atypical in many patients, early diagnosis of IVLBCL remains a challenge in the clinic, and many patients cannot receive proper management in a timely manner. Due to the lack of large-scale prospective studies, the available information about this disease primarily relies on limited case reports. Three different types of variants (classical, cutaneous, and hemophagocytic lymphohistiocytosis (HLH)-associated) have been described (3, 4). Patients with HLH-associated variants display typical clinical HLH, represented by bone marrow (BM) involvement, fever and thrombocytopenia (3). Patients with HLH-associated variants exhibit aggressive onset with poor overall survival (3).

Capillary leak syndrome (CLS) is a rare condition characterized by severe diffuse edema, hypoalbuminemia, and even hypotension (5). The etiology of CLS includes drug-induced, infection-induced, malignancy-associated, and HLH-associated conditions (5, 6). Since 1998, only 8 cases of lymphoma-associated CLS have been reported (7–13). CLS related to HLH has also been documented in a limited number of literature sources (14, 15).

Here, we report a patient with IVLBCL who typically presented with HLH and capillary leak syndrome (CLS), which is a rare but potentially lethal disease. The patient’s condition was complex and dangerous. However, the patient ultimately responded favorably to comprehensive treatment, although the establishment of a diagnosis was challenging.

## Case presentation

A 52-year-old Chinese man with a two-week history of recurrent chills, high fever and fatigue was admitted to our department on July 11, 2016. Physical examination revealed pitting edema in both lower limbs. Laboratory studies revealed anemia (hemoglobin 94 g/L, normal range: 130~175 g/L), thrombocytopenia (platelets 38×10^9^/L, normal range: 100~300×10^9^/L), leukopenia (white blood cells 3.13×10^9^/L, normal range: 3.5~9.5 ×10^9^/L), hypoalbuminemia (albumin 20.7 g/L, normal range: 40~55 g/L), increased levels of serum lactate dehydrogenase (818 U/L, normal range: 110~220 U/L) and ferritin (1212.00 ng/ml, normal range: 13~150 ng/ml). The liver, kidney and thyroid function tests, coagulation studies, and serum folate and vitamin B12 levels were normal. The Coombs test yielded a negative result. Biomarker analyses did not indicate abnormalities suggestive of autoimmune diseases, vasculitis, or malignancies. Plasma Epstein−Barr virus (EBV) DNA was undetectable, and no potentially pathogenic bacteria, viruses, or fungi were identified. Although a weak positive result was obtained from the tuberculosis interferon-γ release assay (TB-IGRA), *Mycobacterium tuberculosis* was not detected. Contrast-enhanced computed tomography revealed only mild pulmonary inflammation, bilateral pleural effusion, and pericardial effusion. No lymphadenopathy or splenomegaly was noted. Initial BM aspirate, flow cytometry, and biopsy examinations did not reveal any significant abnormalities. The patient received empiric antibiotic treatment, accompanied by diuretics and albumin infusion to alleviate edema. Despite a decrease in peak temperature, intermittent fever persisted at 100.9°F (38.3°C) or lower, and there was no improvement in pancytopenia or fatigue. The patient was discharged 25 days after admission with undetermined cause.

He relapsed on August 24, 2016, with a high fever and exacerbated anasarca, prompting emergency admission to a local hospital. He was conscious but exhibited persistent hypotension. Subsequent evaluations revealed bicytopenia (hemoglobin 50 g/L, normal range: 130~175 g/L; platelets 41×10^9^/L, normal range: 100~300×10^9^/L) and severe hypoalbuminemia (16.0 g/L, normal range: 40~55 g/L), with normal cardiac, liver and renal function. An elevated procalcitonin level (1.71 ng/mL, normal range: <0.046 ng/ml) suggested the possibility of septic shock. Empirical broad-spectrum antibiotic therapy was administered, along with fluid resuscitation, multiple albumin infusions, and various vasopressors (dopamine, metaraminol, and norepinephrine). However, his condition did not improve. Routine examinations did not reveal evidence of microbiological infection, autoimmune disease or tumors. Notably, a second BM examination indicated the presence of a few hemophagocytes. Due to persistent recurrent high fever, the patient was transferred to the infectious disease department of our hospital on September 27, 2016.

**Figure 1:**
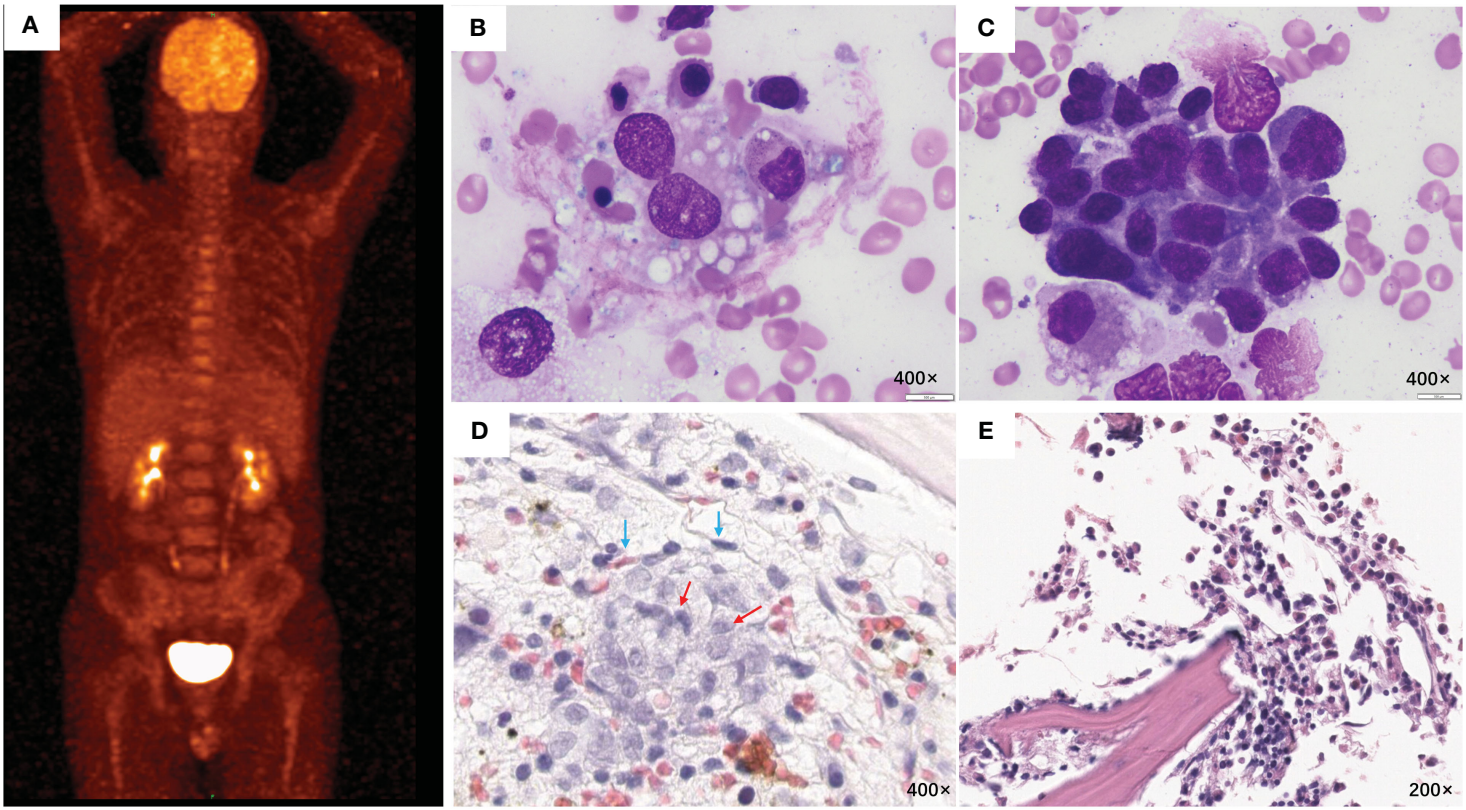
PET/CT and BM examination. **(A)** PET/CT scanning before chemotherapy showed no signs of tumor. **(B)** Many hemophagocytes and **(C)** abnormal lymphocytes were observed in the 6^th^ BM smear (H&E stain, 400×). **(D)** The 6^th^ BM biopsy (H&E stain, 400×) showed intravascular (endothelial cells; blue arrows) aggregation of clustered or cord-like medium-sized atypical cells (red arrows). Immunohistochemical staining was positive for CD20, CD79a, BCL-6, and Mum-1 and negative for CD3, CD10, and EBER1/2-ISH. **(E)** The last BM examination, which was conducted on December 13, 2019, revealed no abnormal cell infiltration. BM, bone marrow; H&E, hematoxylin and eosin.

Subsequent laboratory findings on admission met the HLH-2004 criteria: bicytopenia (hemoglobin 78g/L, normal range: 130~175 g/L; platelets 37×10^9^/L, normal range: 100~300×10^9^/L), decreased fibrinogen (1.12 g/L, normal range: 2~4 g/L), elevated ferritin (1198.00 ng/ml, normal range: 13~150 ng/ml) and soluble interleukin-2 receptor (sIL-R2, >7500 U/ml, normal range: 223~710 U/ml), and hemophagocytosis in the bone marrow. Glucocorticoids were administered continuously to mitigate inflammation. A positron emission tomography/computed tomography (PET/CT) scan revealed minor bilateral pleural and pericardial effusion and mild splenomegaly without significant hypermetabolism (**Figure 1A**). The third BM evaluation did not indicate lymphoma or any specific infection. Diagnostic antituberculosis therapy was initiated due to a weak positive TB-IGRA result but discontinued after 6 weeks due to recurrent high fevers. Despite numerous albumin infusions and diuretic therapy, systemic edema persisted, with serum albumin levels fluctuating between 22.5 and 32.2 g/L (normal range: 40~55 g/L). The fourth BM analysis on October 21, 2016, revealed mild hemophagocytes. In addition, BM culture yielded a positive finding for Staphylococcus hemolyticus, leading to the administration of vancomycin in combination with glucocorticoids. Despite normalization of vital signs and resolution of edema, suspicion of an underlying hematological malignancy persisted, leading to the patient being referred to our department on November 2, 2016.

On November 6, 2016, he experienced a recurrent fever, along with mild erythema on the face, neck, chest, and palms. He also had dyspnea and hypoxemia, with an oxygen saturation level of 88%. Subsequently, he developed an elevated fever, cough, yellow purulent sputum, and hemoptysis. No signs or symptoms of the central nervous system were observed. Enhanced magnetic resonance imaging (MRI) of the brain showed no obvious abnormalities (**Supplementary Figure 1**). The fifth BM examination on November 8, 2016, showed a negative bacterial culture and no significant findings. Mild proteinuria (0.64 g/24 h) and weakly positive KAP monoclonal protein were detected in the urine, but no monoclonal protein was detected in the serum. BNP and myocardial marker levels were normal. After experiencing refractory high fever, hypotension (blood pressure: 74 ~ 85/40 ~ 55 mmHg), and hypoalbuminemia(21.3 g/L, normal range: 40~55 g/L), the patient’s condition rapidly deteriorated, with progressive weakness, hypoxemia, tachycardia, anasarca, splenomegaly and abdominal distension. Laboratory analysis revealed progressive pancytopenia (white blood cells 2.92×10^9^/L, normal range: 3.5~9.5×10^9^/L hemoglobin 60g/L, normal range: 130~175 g/L; platelets 16×10^9^/L, normal range: 100~300×10^9^/L), elevated ferritin (4448.00 ng/ml, normal range: 13~150 ng/ml) and sIL-R2 (>7500 U/ml, normal range: 223~710 U/ml). A diagnosis of HLH complicated with CLS was established. Lymphoma was strongly suspected as the trigger, although conclusive evidence was still lacking. Treatment with an etoposide and dexamethasone (ED) regimen for HLH was initiated in combination with hydroxyethyl starch-based artificial colloid, with a focus on previously overlooked CLSs. Despite one week of stabilization, he experienced a recurrence of critical illness. Notably, numerous telangiectasias were subsequently observed on the erythematous skin of the anterior chest and lower abdominal wall, which later spread to the bilateral ears, shoulders, limbs, and thighs. A deep skin biopsy was recommended but was impeded by severe skin edema and poor performance status. Consequently, the sixth BM examination, which was conducted on December 6, 2016, revealed a large number of hemophagocytes and intravascular aggregation of medium-sized atypical lymphocytes (**Figures 1B−D**) that were positive for CD20, CD79a, BCL-6, and Mum-1 but negative for CD3, CD10, and EBER1/2-ISH, ultimately resulting in a diagnosis of IVLBCL.

After receiving 600 mg of rituximab, the patient exhibited marked improvement, with normalization of vital signs and notable reductions in fatigue, edema and skin lesions. Subsequent CHOP (cyclophosphamide, hydroxydaunorubicin, vincristine and prednisolone) chemotherapy led to the complete resolution of symptoms, signs and laboratory abnormalities. He received a total of 7 cycles of R-CHOP and two intrathecal chemotherapies (methotrexate, cytarabine, and dexamethasone). A post-treatment PET/CT scan showed no signs of tumors. No BM infiltration or central nervous system involvement was detected. Subsequently, he underwent autologous peripheral stem-cell transplantation (ASCT) with the chidamide, cladribine, gemcitabine, and busulfan (ChiCGB) conditioning regimen. The latest BM examination, which was conducted on December 13, 2019, revealed no abnormal cell infiltration (**Figure 1E**). As of the publication date, he has remained disease-free survival for more than 6 years.

**Figure 2:**
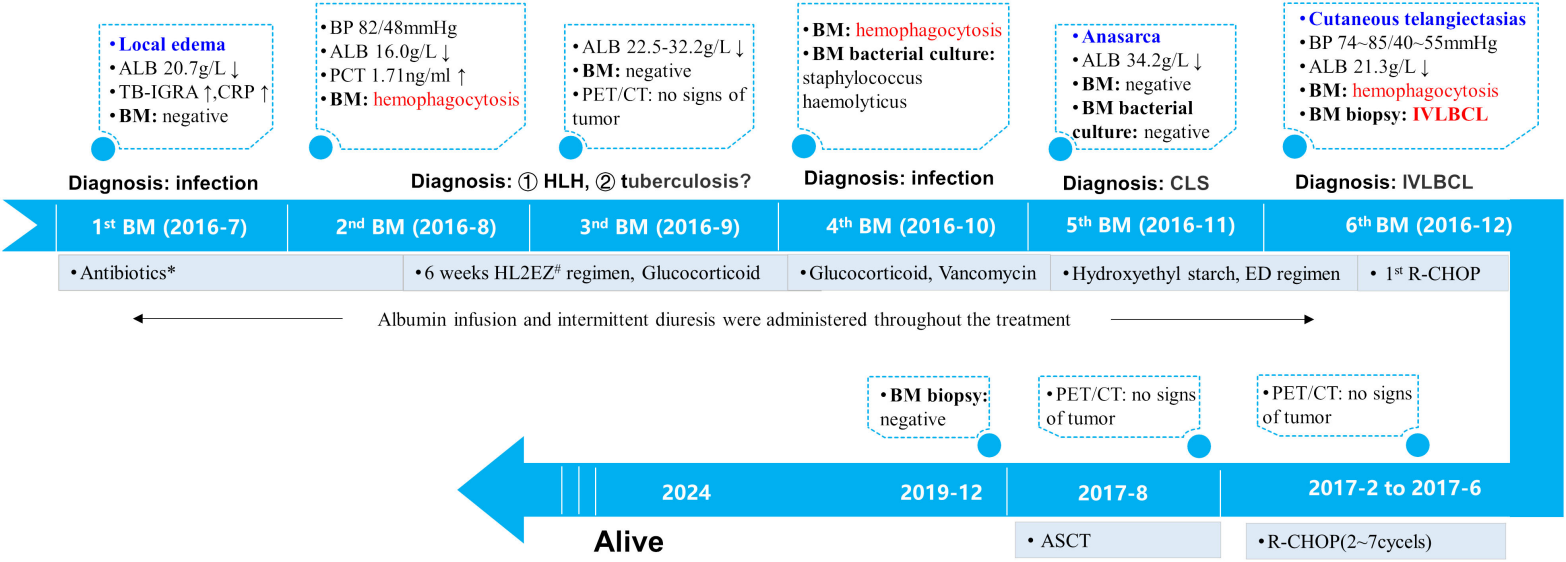
Treatment timeline. The patient presented with recurrent fever, limb edema, hypoalbuminemia, hypotension and cytopenia in July 2016. His inflammatory markers were slightly elevated while TB-IGRA yielded positive results. Therefore, infectious diseases were first considered. Empirical broad-spectrum antibiotic therapy and diagnostic antituberculosis therapy were administered and led to little efficacy.Subsequent laboratory findings confirmed the diagnosis of HLH. Glucocorticoids were given to mitigate inflammation. The 4^th^ BM examination in October 2016 yielded a positive bacterial culture for Staphylococcus hemolyticus and vancomycin was administered. Subsequently, localized edema developed into anasarca. CLS was established. In addition to the ED regimen for HLH, treatment with hydroxyethyl starch-based artificial colloid was initiated to treat CLS. In December 2016, numerous telangiectasias occurred on the erythematous skin. The 6^th^ BM examination confirmed the diagnosis of IVLBCL. Immunochemotherapy with R-CHOP was administered and resulted in prompt alleviation of symptoms, signs and laboratory abnormalities. During hospitalization, the patient received multiple albumin infusions and diuretic therapy. A total of 7 cycles of R-CHOP followed by ASCT were administered, and PET/CT scanning revealed no signs of tumor during mid-treatment or before ASCT. At the time of this publication, he had maintained disease-free survival for more than 6 years. *The patient was treated with cefoperazone/sulbactam, imipenem/cilastatin, levofloxacin and moxifloxacin for anti-infection. #Including isoniazid, levofloxacin, ethambutol, and pyrazinamide. ALB, serum albumin; BM, bone marrow; BP, blood pressure; TB-IGRA: tuberculosis interferon-γ release assay; CRP: c-reactive protein; PCT: procalcitonin; HLH, hemophagocytic lymphohistiocytosis; CLS, capillary leak syndrome; IVLBCL, intravascular large B-cell lymphoma; ED, etoposide and dexamethasone; R-CHOP, rituximab, cyclophosphamide, doxorubicin, vincristine and prednisone; ASCT, autologous peripheral stem-cell transplantation.

The diagnostic and treatment history of this patient is illustrated in **Figure 2**.

## Discussion

The clinical manifestations of IVLBCL are highly variable and nonspecific in most cases. Our patient presented with fever, edema, refractory hypoproteinemia, and hypotension as initial clinical features. Laboratory tests suggested infectious disease, which could not explain all of the patient’s symptoms. Mild CLS might have already been present at that time. Later, positive urine monoclonal protein and numerous cutaneous telangiectasia changes led to a strong suspicion of IVLBCL. Skin lesions are characteristic manifestations of IVLBCL (4, 16). A case of IVLBCL manifested by diffuse telangiectasias has been reported (17). The presence of fever of unknown origin accompanied by CLS and cutaneous manifestations represents a possibility for IVLBCL. Multiple skin or BM biopsies may be a potential method for the diagnosis of IVLBCL.

The standard treatment for IVLBCL has not been defined. The combination of R-CHOP with high-dose methotrexate plus intrathecal chemotherapy has shown safety and efficacy for newly diagnosed IVLBCL patients without obvious central nervous system involvement (18). The positive effects of rituximab can be attributed to its high drug bioavailability and increased complement concentration in small blood vessels (3). Compared with R-CHOP alone, sequential ASCT has been shown to improve IVLBCL (19). After identifying IVLBCL as the underlying disease, the patient’s treatment plan was promptly adjusted to target lymphoma, and the R-CHOP regimen for IVLBCL resulted in rapid alleviation of CLS and HLH symptoms. Subsequently, the patient underwent intrathecal chemotherapy and ASCT. Although the patient did not receive high-dose methotrexate, the long-term survival data indicated that the combination of R-CHOP and ASCT is feasible for eradicating IVLBCL.

We present a case of IVLBCL complicated with HLH and CLS, which has not been previously reported. The pathogenesis of CLS involves hyperpermeability and transient endothelial dysfunction, which causes the leakage of a significant amount of plasma proteins and body fluids from blood vessels into the tissue space (5, 20). Multiple factors, including cytokines/chemokines, adhesion molecules, angiogenic factors, and autoantibodies, contribute to the development of CLS (5). Hypercytokinemia is believed to be the underlying cause of CLS (6). The cytokines interleukin-2 (IL-2) and vascular endothelial growth factor (VEGF) may play essential roles in endothelial hyperpermeability (5). Abnormal cytokine levels are correlated with abnormal immune function. Impaired capillary endothelium and increased vascular permeability are caused by various diseases (6). For CLS etiology, 61.3% of CLS patients had hematologic malignancies, while non-Hodgkin’s lymphoma was the most prevalent disease (5). Cytokines are crucial for interactions between the immune system and the host across various hematologic tumors.

To our knowledge, here. A literature search in PubMed with the key words ‘lymphoma’ and ‘CLS’ revealed very few cases (**Table 1**). Regarding possible triggers, lymphoma-complicated CLS patients have elevated levels of various cytokines, such as granulocyte colony-stimulating factor (G-CSF), interleukin-6 (IL-6), interleukin-10 (IL-10), interferon-gamma (IFN-γ) and tumor necrosis factor alpha (TNF-α) (7, 10–12). Cytokines, such as IL-6 and IL-10, have also been found to play significant roles in IVLBCL (21, 22). HLH-associated variants of IVLBCL (the so-called Asian variant) frequently present with fever, HLH, bone marrow involvement and hypercytokinemia (23). The exact mechanism by which HLH has a secondary etiology is unknown, but immune hyperactivity is a result (6). MAN C et al. reported that 18.1% (47/259) of secondary HLH patients were complicated with CLS (14). Malignancy-associated HLH is also associated with cytokine storms (24). In response to cytokines, vascular permeability increases (20, 25). In addition, the infiltration of lymphatic cancer cells into the vascular lumen (2) is suspected to damage the normal vascular endothelium. Cytokine release and vascular endothelial damage are related to the pathophysiology of CLS (5, 6), which appears to be the core pathophysiological mechanism in HLH-associated IVLBCL variants complicated with CLS.

**Table 1:** Cases of capillary leak syndrome associated with lymphoma in the literature.

References no.	Authors	Sex	Age(years)	Clinical features	Lowest blood pressure (mmHg)	Laboratory data	Classification of lymphoma	Chemotherapy	Other treatments	Outcome
7	Xu Han, et al., 2023	M	60	diffuse abdominal distention, intermittent fever, fatigue, loss of appetite, generalized abdominal distension	50/39	hypoalbuminemia (2.9 g/dl), hyperkalemia (6.4 mmol/l), coagulation dysfunction, renal insufficient, increased LDH, increased ferritin (2305 ug/l), elevated b-2 macroglobulin (14 mg/l)	Diffuse large B-cell lymphoma	RCHOP	fluid infusion, albumin, dopamine and noradrenaline	Died
8	Pothen L, et al.,2014	F	75	hypotension leg edema anasarca	80/40	renal insufficiency, hypoalbuminemia, pancytopenia, decreased fibrinogen	Diffuse large B-cell lymphoma	RCHOP	furosemide, protein perfusion (albumin and geloplasma), theophylline	Alive
9	Laura S. Lourdes, e al.,2012	F	71	lower extremity and facial swelling, nausea, vomiting, diarrhea, enlarged lymph nodes (left inguinal and retroperitoneal)	70/NA	acute renal failure (blood urea nitrogen 51 mg/dL and serum creatinine 4.99 mg/dL), hypoalbuminemia (albumin 3 mg/dL)	ALK-negative anaplastic large cell lymphoma	CHOP	methylprednisolone	Died (ALCL relapse with CNS involvement)
10	Takako Umemoto et al.,2011	M	13	high fever, dyspnea, hypoxemia, pulmonary edema weight gain, intraabdominal bulky lymphadenopathy	NA	leukocytosis, thrombocytopenia, hypoproteinemia, elevated ferritin (1216 mg/dL), hypofibrinogenemia (67 mg/dL)	ALK+ anaplastic large cell lymphoma	etoposide, cytosine arabinoside, dexamethasone	high dose methylprednisolone, high-dose immunoglobulin	Alive
11	E Andres, et al., 2000	F	83	edema	NA	high levels of plasma interleukin (IL) 2 and IL 6	follicular non-Hodgkin’s lymphoma	Unknown	corticoids	Alive
12	Jillella AP, et al., 2000	F	43	edema, fever, weight gain, anemia, thrombocytopenia, lymphadenopathy	NA	hypoalbuminemia (albumin 2.4 mg/dL), elevated TNF-α levels	Diffuse large B-cell lymphoma	CHOP	NA	Alive
12	Jillella AP, et al., 2000	M	32	Edema, fever, pleural effusion, ascites, lymphadenopathy	NA	hypoalbuminemia (albumin 2.0 mg/dL), elevated TNF-α levels	T-cell lymphoma	CHOP	NA	Died
13	Takimoto Y, et al., 1998	M	43	systemic edema, pleural effusion, and ascites.	NA	NA	Large cell lymphoma (γδT cell)	THP-COOP	albumin, diuretics, antihistamines, prednisolone	Alive (Relapse)

R-CHOP, Rituximab, Cyclophosphamide, Doxorubicin, Vincristine and Prednisone. CHOP, Cyclophosphamide, doxorubicin, vincristine and prednisone. EPOCH, etoposide, prednisone, vincristine, cyclophosphamide and doxorubicin. THP-COP, pirarubicin, cyclophosphamide, vindesine and prednisone. NA, not available.

Currently, there are no guidelines for CLS management in the clinic. Since lymphoma complicated with CLS is very rare, disease management might be more empirical than the management of simple CLS. In general, fluid resuscitation (with crystalloids as the initial choice and albumin as the last resort) is necessary while maintaining permissive hypotension. Vasoactive amines can be used for uncontrolled hypotension (5, 26). It has been reported that patients with CLS experience improved symptoms following treatment with albumin and diuresis (15). In this case, albumin and diuretics were administered before the CLS was established. Subsequent to the CLS, hydroxyethyl starch was promptly administered for fluid resuscitation. However, the pure support treatment had little effect. Patients with CLSs and hematologic malignancies have the worst overall survival among those with malignancies associated with CLSs (27). The prognosis of HLH patients complicated with CLSs is markedly inferior to that of patients without CLSs (14, 15). In highly active HLH, disease-adapted HLH-94 with etoposide was administered prior to cancer-specific therapy (28). In our patient, ED was administered to control HLH. Surprisingly, the clinical manifestations of CLS, such as fever and edema, were also transiently relieved, which indicated that HLH and CLS may share the same inflammatory background and underlying disease. Subsequently, immunochemotherapy with R-CHOP led to complete remission of HLH and CLS, which further demonstrated that IVLBCL triggered HLH and CLS. Therefore, treatment of the underlying disease of CLS and HLH plays a critical role compared with pure support therapy.

Limitations associated with this case report warrant mention. However, random skin biopsy is appropriate for the diagnosis of IVLBCL (29). However, the patient’s poor general condition and severe skin edema made it difficult to perform a skin biopsy. Moreover, results for serum IL-10, which is a valuable biomarker for early diagnosis and therapeutic monitoring in IVLBCL, are lacking (21).

In conclusion, the presence of fever of unknown origin accompanied by CLS and cutaneous manifestations represents a possibility for IVLBCL. Multiple skin or bone marrow biopsies facilitate the confirmation of the diagnosis. The combination of R-CHOP and ASCT may represent a curative approach for this lethal disease. Cytokine release and vascular endothelial damage appear to be the core pathophysiological mechanisms in HLH-associated IVLBCL complicated with CLS. Our case provides some insights into the mechanism and clinical management of the concurrent occurrence of IVLBCL complicated with HLH and CLS.

## Data availability statement

The original contributions presented in the study are included in the article/**Supplementary Material**. Further inquiries can be directed to the corresponding authors.

## Ethics statement

Written informed consent was obtained from the individual(s) for the publication of any potentially identifiable images or data included in this article.

## Author contributions

JW: Data curation, Formal analysis, Investigation, Writing – original draft, Writing – review & editing. JX: Formal analysis, Investigation, Writing – original draft, Writing – review & editing. JJ: Investigation, Resources, Writing – review & editing. WZ: Investigation, Resources, Writing – review & editing. QZ: Investigation, Resources, Writing – review & editing. TL: Resources, Writing – review & editing. YZ: Supervision, Writing – review & editing. HM: Conceptualization, Resources, Supervision, Writing – review & editing.

## Supplementary material

Supplementary Figure 1 Brain MRI. Enhanced MRI of the brain revealed no obvious abnormalities. MRI, magnetic resonance imaging **(A, B)**.
