# Supplementary figures and images for: Intravascular large B-cell lymphoma as a covert trigger for hemophagocytic lymphohistiocytosis complicated with capillary leak syndrome: a case report and literature review
Source: Front Immunol. 2024 Jul 12;15:1403376. doi: 10.3389/fimmu.2024.1403376 (PMC11272449; doi:10.3389/fimmu.2024.1403376)

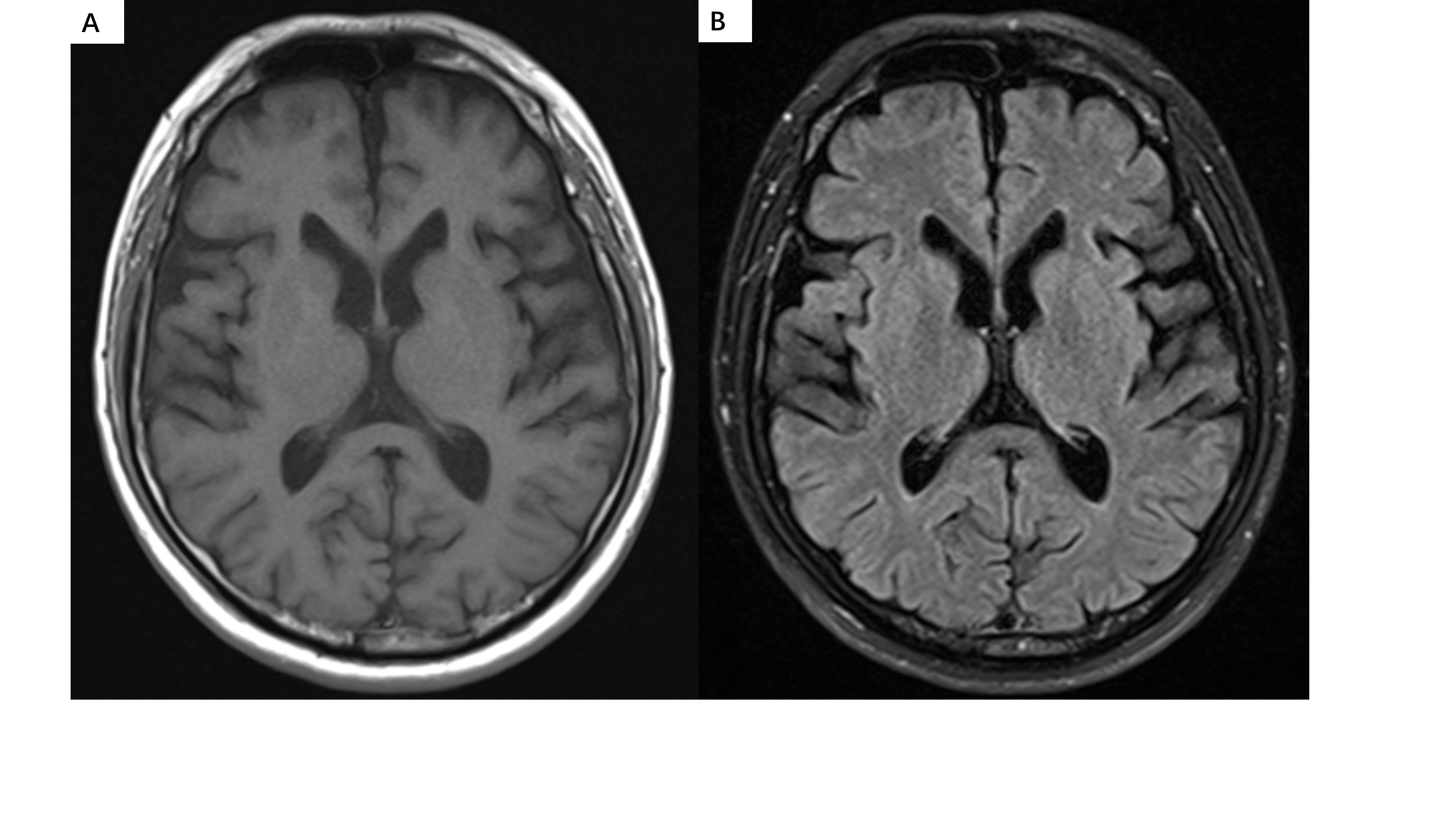

Supplement: Supplementary Figure 1 — Brain MRI. Enhanced MRI of the brain revealed no obvious abnormalities. MRI, magnetic resonance imaging (A, B). [file Image_1.tif]
